# Does misclassification of former tobacco smokers explain the ‘smoker’s paradox’ in the risk of COVID-19? Insights from the Stockholm Public Health Cohort

**DOI:** 10.1177/14034948231174279

**Published:** 2023-05-10

**Authors:** Ahmed N. Shaaban, Filip Andersson, Cecilia Magnusson, Nicola Orsini, Ida H. Caspersen, Sebastian Peña, Sakari Karvonen, Per Magnus, Maria R. Galanti

**Affiliations:** 1 Department of Global Public Health, Karolinska Institutet, Sweden; 2 Centre for Epidemiology and Community Medicine, Stockholm Region, (CES), Sweden; 3 Centre for Fertility and Health, Norwegian Institute of Public Health, Norway; 4 Finnish Institute for Health and Welfare, Finland

**Keywords:** COVID-19, SARS-CoV-2, tobacco smoking, former smokers, misclassification bias, population-based cohort, Sweden

## Abstract

**Background:**

The association between tobacco smoking and the risk of COVID-19 and its adverse outcomes is controversial, as studies reported contrasting findings. Bias due to misclassification of the exposure in the analyses of current versus non-current smoking could be a possible explanation because former smokers may have higher background risks of the disease due to co-morbidity. The aim of the study was to investigate the extent of this potential bias by separating non-, former, and current smokers when assessing the risk or prognosis of diseases.

**Methods:**

We analysed data from 43,400 participants in the Stockholm Public Health Cohort, Sweden, with information on smoking obtained prior to the pandemic. We estimated the risk of COVID-19, hospital admissions and death for (a) former and current smokers relative to non-smokers, (b) current smokers relative to non-current smokers, that is, including former smokers; adjusting for potential confounders (aRR).

**Results:**

The aRR of a COVID-19 diagnosis was elevated for former smokers compared with non-smokers (1.07; 95% confidence interval (CI) =1.00–1.15); including hospital admission with any COVID-19 diagnosis (aRR= 1.23; 95% CI = 1.03–1.48); or with COVID-19 as the main diagnosis (aRR=1.23, 95% CI= 1.01–1.49); and death within 30 days with COVID-19 as the main or a contributory cause (aRR=1.40; 95% CI=1.00–1.95). Current smoking was negatively associated with risk of COVID-19 (aRR=0.79; 95% CI=0.68–0.91).

**Conclusions:**

**Separating non-smokers from former smokers when assessing the disease risk or prognosis is essential to avoid bias. However, the negative association between current smoking and the risk of COVID-19 could not be entirely explained by misclassification.**

## Introduction

The coronavirus disease (COVID-19) pandemic, caused by severe acute respiratory syndrome coronavirus 2 (SARS-CoV-2), has caused more than 605 million confirmed cases of COVID-19 and about 6.5 million deaths around the world by 12 September 2022 [1]. Tobacco smoking has been investigated as a potential risk factor for the disease. The association between tobacco smoking and SARS-CoV-2 infection or the disease’s adverse outcomes remains controversial, as studies reported mixed findings. Some studies have reported a negative association between smoking and COVID-19 infection [2] or hospitalisations due to COVID-19 [3,4], yet others have shown a positive association [5,6].

It has been suggested that the observations of a negative association between tobacco smoking and COVID-19 may result from biases, including confounding, selection or bias due to misclassification of the exposure. This latter bias may be present, for instance, when the exposure is defined as current versus non-current smokers (therefore including former and never smoker in the same reference category). Smokers who develop smoking-related chronic diseases are more likely to quit smoking than smokers without these conditions [7]. For instance, smokers with smoking-related diseases were found to have an increased motivation to quit smoking due to the direct negative effect of smoking, such as worsening symptoms or reduced quality of life [8]. Moreover, these individuals often receive feedback from healthcare providers about their lung function, biomarkers feedback related to their smoking behaviour, and the impact of smoking on their disease progression [9]. Healthcare providers can also help smokers understand the benefits of smoking cessation, such as improved disease management and lower risk of disease progression or complications [10].

The presence of such morbidity may, in turn, increase the risk of a SARS-CoV-2 infection and/or its adverse prognosis. The risk of infection among smokers may be enhanced by several biological or behavioural factors. First, smoking-related co-morbidity may imply accelerated lung function decline [11], and this decline is associated with infection susceptibility, inflammation and impaired immunity [11,12]. Also, some tobacco-related diseases, such as chronic obstructive pulmonary disease (COPD), do not reverse after smoking cessation [11]. Studies have shown that former smokers with COPD tend to have a similar number and type of inflammatory cells as current smokers, and this indicates that inflammation is ongoing even after smoking cessation [13]. In fact, a recent systematic review found that former smokers have a somewhat increased risk of hospitalisation with COVID-19 as well as an enhanced disease severity [2].

In Sweden, the transition to smokeless tobacco use (‘*snus*’) has been very common among men quitting smoking [14], and it can be hypothesised that *snus* users also have a high risk of infections of the airways due to frequent contact between the hands and the oral mucosa. Also, unlike smokers, *snus* users are not subjected to restrictions on tobacco use in proximity to others, which can have unfavourable consequences on scoial distancing during the pandemic.

For the reasons above, analyses including ‘former’ in the same reference group as ‘never’ smokers when estimating the relative risk of COVID-19 among ‘current smokers’ may yield downwards biased estimates of associations, reported in previous studies based on such a comparison [15–17].

The aim of this study was to assess the presence and entity of this potential bias within the same study, drawing on longitudinal data from the Stockholm Public Health Cohort (SPHC). For this purpose, we explored the relative risk of COVID-19 and its adverse outcomes comparing (a) current and former smoking contrasted to non-smokers; (b) current smokers contrasted to non-current smokers, where the reference group includes both never and former smokers.

## Methods

### Study design and data sources

The study was based on longitudinal data collected within the framework of the SPHC. The SPHC is a population-based cohort that had been established within the region of Stockholm, with participants recruited in subsequent surveys conducted in 2002, 2006, 2010 and 2014. For the purpose of this study, we used information collected during the 2010 and 2014 surveys. Data were collected using postal or web-based questionnaires covering health-related and lifestyle information, including tobacco use. Self-reported information has been complemented by information from healthcare and socio-demographic registries.

The data collection was managed by Statistics Sweden in collaboration with the Department of Public Health Sciences at Karolinska Institute [18]. The cohort profile, including the questionnaire, has been described in detail elsewhere [18]. Socio-demographic information (sex, age, achieved education, occupational risk for infection, income, cohabitation and country of birth) was extracted through record-linkage with the register of the total population of the region of Stockholm held by Statistics Sweden. We used the national personal identification number assigned to every resident in Sweden at birth or at immigration to obtain information on diagnoses of COVID-19 among individuals in this cohort through record-linkage with the regional database of inpatient and outpatient health care (VAL database).

### Participants

**Figure 1.**
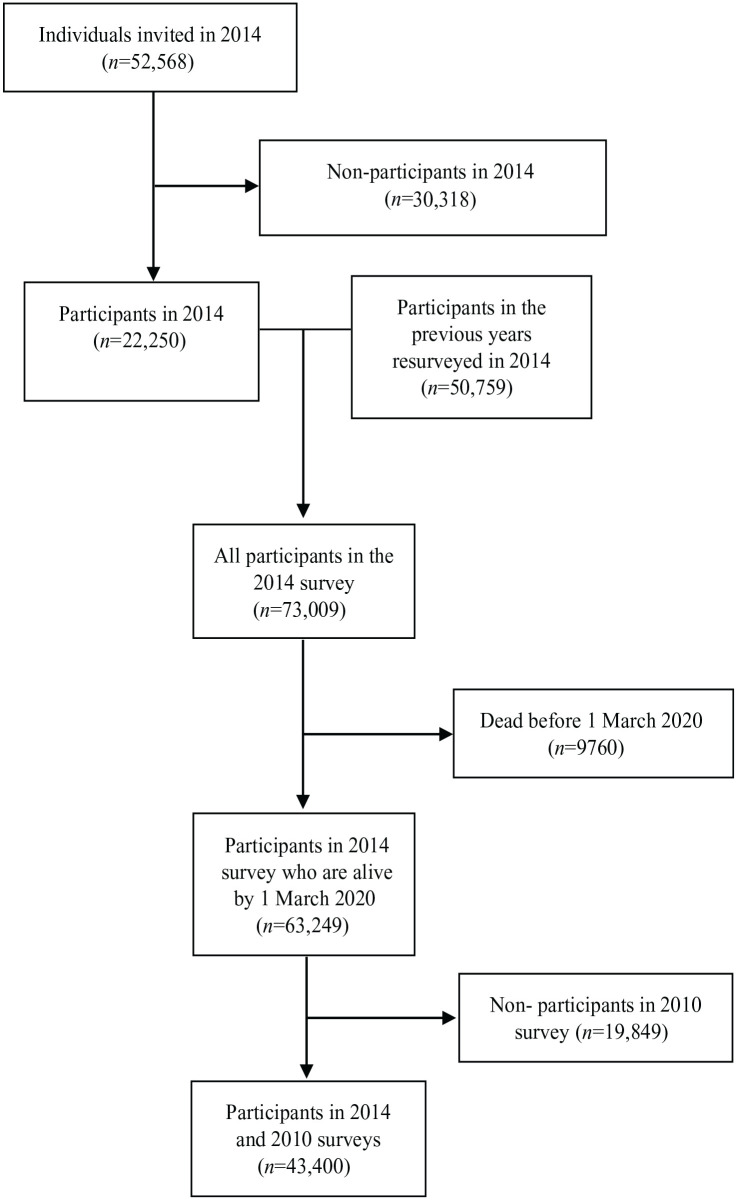
Flow diagram of invited individuals and participants.

The derivation of the study sample is shown in Figure 1.

### Exposure

Participants were initially grouped according to their self-reported tobacco smoking status in 2014, whereby they were required to answer the following questions: A. Have you ever smoked tobacco daily, or almost daily, for at least six months? (1: Yes, 2: No); B. Do you currently smoke tobacco daily or almost daily? (1: Yes, 2: No). For the purpose of this analysis, we first grouped the answers into three categories: non-smoker (if A=2); current smoker (if A=1, B=1); former smoker (if A= 1, B= 2). Smoking status in 2010 was then used to refine this latter category to also include former smokers, those who reported not smoking in 2014 but reported former or current daily smoking in 2010. To assess the potential bias induced by the inclusion of former smokers in the category of non-current smokers, we then re-categorised the answers into a dichotomous variable ‘Current non-smoker’ (if A=2 or if A= 1, B= 2); and ‘Current smoker’ (if A=1, B=1).

### Outcome

Diagnoses were included between 1 March 2020 and 31 August 2021 (date of last update of record linkage). The rollout of COVID-19 vaccinations started in Sweden in January 2021. Five binary outcomes (Yes/No) were included in the analysis:

1. Any diagnosis of COVID-19, whether in a hospital or outside, consisting of at least a positive polymerase chain reaction (PCR) test reported by the laboratories to Sweden’s national electronic surveillance system for communicable diseases, SmiNet.
2. Hospital admission with a diagnosis of COVID-19 (ICD-10 codes U071 and U072) registered either as main or as a concomitant diagnosis.
3. Admission to an intensive care unit (ICU) with a diagnosis of COVID-19 (ICD-10 codes as above).
4. Death by COVID-19, established using the Swedish Cause of Death Registry, which is based on the death certificate filled in by physicians. All deaths occurring during the follow-up period with COVID-19 registered as the main cause were included. The restriction to the main cause of death was done to maximise the specificity of the diagnosis [19].
5. Death within 30 days from a diagnosis of COVID-19, listed as either the main or a contributory cause. This latter analysis was conducted for sensitivity purposes.

### Confounders

Adjustments were made for potential confounders, selected using a directed acyclic graph (Supplemental material Figure S1 online). Socio-demographic information (sex, age, achieved education, occupational risk for infection, income, cohabitation and country of birth) was extracted through record-linkage with the register of the total population of the region of Stockholm held by Statistics Sweden. These covariates were categorised as follows: Sex (male/female), Age in years (continuous), Education (compulsory school, i.e. nine years of schooling; high school, i.e. two or three years of schooling after the compulsory education; university; unknown); Disposable yearly income in Swedish crowns (continuous); Cohabitation with others (Yes/No); Country of birth (Sweden; Other Nordic country; Other country). Occupational risk for infection with SARS-CoV-2 was categorised as ‘high’, ‘moderate’ or ‘low’ based on a priori knowledge of exposure to the transmission of the virus.

Three variables were derived from self-reports in the SPHC in 2010 and 2014: a diagnosis of sexually transmitted diseases in the past 12 months; diagnoses of chronic diseases; and the use of *snus*.

We used ‘sexually transmitted diseases’ as a proxy for risky social contact exposure to SARS-CoV-2 transmission. In fact, risky sexual behaviours, such as unprotected sex and multi-partnership, are causally linked to sexually transmitted diseases and correlate heavily with smoking [20,21]. This information and the presence of chronic diseases were self-reported as yes/no. *Snus* use, categorised as non-use; former use; current use, was derived in the same way as smoking.

### Statistical analysis

Risk ratios (RRs) and their corresponding 95% confidence intervals (CIs) for COVID-19 infection, hospitalisation, ICU or death due to COVID-19 were estimated through generalised linear models. We specified the Poisson family for the binary health outcomes with robust standard errors estimated using maximum likelihood [22]. We ran two sets of analyses. In the main analysis, we derived the risk of the selected outcomes between current and former smokers relative to non-smokers. Additionally, we compared the risk of the selected outcomes between current- and non-current smokers, where this latter category included former smokers. We expected that the inclusion of former smokers in the reference group would increase the absolute risk in this group, resulting in RR for current smokers further from the null in the case of a negative association and closer to the null in the case of a positive association, compared with the estimates obtained in the main analysis [23].

Adjustments were made for the putative confounders listed above: sex, age, cohabitation, education, income, occupational risk for infection, country of birth, *snus* use, existence of sexually transmitted or chronic diseases as covariates in the study. Missing data in any co-variate (less than 10%) were considered ignorable (complete case analysis). All analyses were conducted using STATA®, version 17 (StataCorp LP, College Station, Texas, USA).

## Results

A total of 43,400 participants (39,425 with complete data) were included in the study. The derivation of the study sample is shown in a flowchart (Figure 1). Table I shows the baseline socio-demographic characteristics and incident diagnoses of COVID-19 among the cohort participants, separately by categories of smoking behaviour.

**Table I.** Characteristics of participants of the 2010–2014 Stockholm Public Health Cohort stratified by smoking behaviour (*N*= 43,400).

	All	Non-smokers	Current smokers	Former smokers
	*N*= 43,400	*n*= 22,452	*n*= 3263	*n*=3263
	*n* (%)	*n* (%)	*n* (%)	*n* (%)
**Sex**				
Male	18,326 (42.2)	13,074 (58.2)	2036 (62.4)	9493 (56.5)
Female	25,074 (57.8)	9378 (41.8)	1227 (37.6)	7409 (43.8)
**Age at the beginning of follow-up (1 March 2020)**, years, mean (SD)	62.67 (15.0)	59.8 (15.7)	63.1 (13.0)	66.0 (13.5)
**Education**				
Compulsory (⩽9 years)	4753 (11.0)	1761 (7.9)	614 (19.0)	2193 (13.0)
High school (10–12 years)	16,110 (37.3)	7260 (32.5)	1596 (49.4)	6958 (41.4)
University (> 12 years)	22,297 (51.7)	13,316 (59.6)	1021 (31.6)	7668 (45.6)
**Occupational risk for infection**				
Low	31,350 (76.8)	15,945 (75.9)	2021 (67.9)	12,749 (79.1)
Moderate	5487 (13.4)	2979 (14.2)	539 (18.1)	1906 (11.8)
High	3996 (9.8)	2073 (9.9)	415 (13.9)	1453 (9.0)
**Disposable income,** per 1000 Swedish crowns/year, mean (SD)	3078.041 (5138.666)	3185.405 (6035.701)	2495.946 (3290.388)	3075.314 (4128.688)
**Cohabitation**				
Yes	32,310 (74.5)	17,289 (77.3)	2075 (64.1)	12,439 (73.9)
No	10,936 (25.5)	5092 (22.7)	1164 (35.9)	4405 (26.1)
**Country of birth**				
Sweden	37,018 (85.3)	19,282 (85.9)	2595 (79.5)	14,538 (86.0)
Other Nordic countries	2213 (5.1)	955 (4.2)	230 (7.1)	975 (5.8)
Other countries	4169 (9.6)	2215 (9.9)	438 (13.4)	1389 (8.2)
**Sexually transmitted diseases**				
No	42,453 (97.8)	21,945 (97.9)	3189 (97.9)	16,564 (98.2)
Yes	861 (2.0)	471 (2.1)	67 (2.1)	310 (1.8)
**Chronic diseases** ^a^				
No	30,704 (70.7)	17,292 (77.0)	2096 (64.2)	10,858 (64.2)
Yes	12,696 (29.3)	5160 (22.0)	1167 (35.8)	6.044 (35.8)
***Snus* use**				
Non-*snus* user	35,951 (84.2)	20,151 (91.1)	2804 (86.6)	12,551 (74.8)
Current *snus* user	3412 (8.0)	1055 (4.8)	261 (8.1)	2055 (12.2)
Former *snus* user	3308 (7.7)	923 (4.2)	171 (5.3)	2182 (13.0)

The sum of the total in the cells might differ from the total because of missing information.

*Snus* = smokeless tobacco use.

a Self-report of chronic diseases.

The proportion of current smokers in this sample was 7.5%, and that of former smokers was 38.9%. Former smokers were older compared with both non-smokers and current smokers. The proportion of chronic diseases self-reported by current and former smokers (35.8%) was similar, and it was higher than among non-smokers (22.0%). The proportion reporting sexually transmitted diseases in the past 12 months was similar in all groups of smoking behaviour. Current *snus* use was more frequent among former smokers (12.2%) compared with non-smokers and current smokers (4.8 % and 8.1%, respectively). The cumulative incidence of COVID-19 diagnoses was 9.1%, and that of death was 0.43%. Table II reports the distribution of incident COVID-19 diagnoses and related outcomes (hospital admissions, admission to ICU, and death) across categories of smoking status.

**Table II.** Diagnosis of COVID-19 and its adverse outcomes by smoking status (%).

	Diagnoses of COVID-19		Hospital admission with COVID-19 diagnosis		Hospital admission with COVID-19 as the main diagnosis		Intensive care		Death due to COVID-19 as the main cause		Death within 30 days with COVID-19 as the main or a contributory cause	
	No	Yes	No	Yes	No	Yes	No	Yes	No	Yes	No	Yes
**Tobacco smoking**												
Non-smokers	20,281 (52.3)	2171 (56.2)	22,200 (52.8)	252 (43.2)	22,238 (52.8)	214 (43.4)	22,430 (52.7)	33 (43.1)	22,384 (52.7)	68 (42.8)	22,381 (52.7)	71 (41.8)
Current smokers	3043 (7.8)	220 (5.7)	3221 (7.7)	42 (7.2)	3230 (7.7)	33 (6.7)	3260 (7.7)	3 (5.9)	3253 (7.7)	10 (6.3)	3251 (7.7)	12 (7.1)
Former smokers	15,751 (39.8)	1475 (38.1)	16,613 (39.5)	289 (49.6)	16,656 (39.5)	246 (49.9)	16,876 (39.7)	26 (51.0)	16,821 (39.6)	81 (50.9)	16,815 (39.6)	87 (51.2)

The risk of being diagnosed with COVID-19 between 1 March 2020 and 31 August 2021 was higher among former smokers than among non-smokers (adjusted RR 1.07, 95% CI =1.00–1.15) (Table III). The risk of being hospitalised with any diagnosis of COVID-19 (adjusted RR 1.23, 95% CI =1.03–1.48); with COVID-19 as the main diagnosis (adjusted RR 1.22, 95% CI =1.00–1.49); as well as the risk of death with COVID-19 within 30 days regardless of the cause (adjusted RR 1.40, 95% CI =1.00–1.95) was also higher among former smokers compared with non-smokers (Table III). The risk of being diagnosed with COVID-19 was lower for current smokers compared with non-smokers (adjusted RR 0.79, 95% CI =0.68–0.91), while the risks of hospital admission, admission to intensive care and of death did not differ from those of non-smokers (Table III).

**Table III.** Adjusted risk ratios (RRs) and 95% confidence intervals (CIs) of COVID-19 diagnosis and adverse outcomes for former or current smokers compared with non-smokers.

	Diagnoses of COVID-19	Hospital admission with COVID-19 diagnosis	Hospital admission with COVID-19 as the main diagnosis	Intensive care	Death due to COVID-19 as the main cause	Death within 30 days with COVID-19 as the main or a contributory cause
	Adjusted^a^ RR	Adjusted^a^ RR	Adjusted^a^ RR	Adjusted^a^ RR	Adjusted^a^ RR	Adjusted^a^ RR
	(95% CI)	(95% CI)	(95% CI)	(95% CI)	(95% CI)	(95% CI)
**Tobacco smoking**						
Non-smokers	(ref.)	(ref.)	(ref.)	(ref.)	(ref.)	(ref.)
Current smokers	0.79 (0.68–0.91)	1.02 (0.72–1.44)	0.92 (0.62–1.35)	0.89 (0.27–2.89)	1.28 (0.63–2.62)	1.44 (0.76–2.75)
Former smokers	1.07 (1.00–1.15)	1.23 (1.03–1.48)	1.23 (1.01–1.49)	1.31 (0.72–2.37)	1.37 (0.97–1.93)	1.40 (1.00–1.95)

a Adjusted for sex, age, education, income, occupational risk, country of birth, cohabitation, *snus* use, sexually transmitted diseases and chronic diseases.

*Snus* = smokeless tobacco use.

ref.: reference

Table IV shows the risk of COVID-19 and related disease outcomes among current smokers compared with current non-smokers, that is, a reference group including both non- and former smokers. The risk of infection was significantly lower among current smokers (adjusted RR 0.76, 95% CI= 0.67–0.87) than among non-current smokers, while again, the estimated relative risk of hospitalisation, ICU and death was compatible with no association.

**Table IV.** Adjusted risk ratios (RRs) and 95% confidence intervals (CIs) of COVID-19 diagnosis and adverse outcomes for current smokers compared with non- or former smokers (non-current smokers).

	Diagnoses of COVID-19	Hospital admission with COVID-19 diagnosis	Hospital admission with COVID-19 as the main diagnosis	Intensive care	Death due to COVID-19 as the main cause	Death within 30 days with COVID-19 as the main or a contributory cause
	Adjusted^a^ RR	Adjusted^a^ RR	Adjusted^a^ RR	Adjusted^a^ RR	Adjusted^a^ RR	Adjusted^a^ RR
	(95% CI)	(95% CI)	(95% CI)	(95% CI)	(95% CI)	(95% CI)
**Tobacco smoking**						
Non- or former smokers	(ref.)	(ref.)	(ref.)	(ref.)	(ref.)	(ref.)
Current smokers	0.76 (0.67–0.87)	0.91 (0.66–1.27)	0.82 (0.57–1.20)	0.77 (0.24–2.47)	1.08 (0.54–2.13)	1.20 (0.65–2.21)

a Adjusted for sex, age, education, income, occupational risk, country of birth, cohabitation, *snus* use, sexually transmitted diseases and chronic diseases.

*Snus* = smokeless tobacco use.

ref.: reference

## Discussion

In this population-based cohort, we examined the prospective association between tobacco smoking and SARS-CoV-2 infection risk and its adverse outcomes six years later, looking for evidence of potential bias introduced by the comparison between current and non-current smoking. The data allowed a refined definition of non-, former and current daily smoking, thus enabling the comparison between results potentially biased because they were obtained with the use of a misclassified reference group and those obtained with a correctly specified reference group. Former smoking was associated with a higher risk of COVID-19 infection, hospitalisation, and death compared with non-smoking, even after considering the presence of chronic diseases. On the other hand, current tobacco smoking was associated with a lower risk of COVID-19 infection after controlling for potential confounders. Our study showed that the results based on the misclassified comparison (categorising former smokers as non-smokers) were similar, but the negative association between current smoking and infection appeared to be stronger, thus speaking for a moderate amount of downward bias (about 4%).

The results from this study are in line with previous reports of a higher risk of COVID-19 infection and adverse outcomes among former smokers when compared with non-smokers [3,24]. Also, they are in line with those studies showing a higher risk of COVID-19 disease severity among former but not among current smokers [2,25] compared with non-smokers.

Previous studies indicate that former smokers are more likely to be older and have smoked for a longer time than current smokers or suffer more comorbidities when compared with non- or current smokers [26–28]. This may indicate that former smokers are more likely to suffer from COVID-19 infection and adverse disease outcomes, given their pre-existing tobacco-related comorbidities. These observed differences in outcomes confirm the relevance of accurately separating former from non-smokers when investigating the association between smoking and the risk or prognosis of COVID-19 and potentially also of other diseases. Results from previous studies that misclassified former smokers as current non-smokers due to incomplete data or inconsistent assessment of smoking status should be handled with caution. If former smokers are at higher risk due to co-morbidity, including them in the same category as non-smokers may spuriously inflate the risk of this latter group and result in biased estimates of the association between current smoking and the disease, as this study confirms. In fact, the moderate amount of bias estimated in this sample may not be assumed in other studies based on samples with different behavioural or socio-demographic characteristics.

Current smokers were at lower risk of infection with SARS-CoV-2 in this study, irrespective of smoking categorisation, while the prognostic outcomes of a diagnosis of COVID-19 among smokers in this study were not different from those of non-smokers. The negative association between current smoking and the risk of COVID-19 in our study could not be entirely explained by the misclassification of the exposure. Other studies also reported a lower risk of infection among smokers [2], in contrast with the expected deleterious effect of smoking due to its well-established causal role in respiratory tract diseases and infections [29]. A convincing explanation of this puzzling association is still lacking. Hypotheses have been forwarded, for instance, regarding the potential protective role of nicotine either as an anti-inflammatory agent or due to its ability to bind to the ACE2 cell-membrane protein, the entry point for the SARS-CoV-2 virus, hence blocking the virus from binding to the protein [30,31]. However, recent population-based studies conducted in Sweden and Finland showed that the users of smokeless tobacco *snus* are at a higher risk of infection compared with non-users [32,33]. Because *snus* delivers high amounts of nicotine, these results did not support a protective role of this substance. It is also possible that our study may not have accounted for all confounding factors that could influence the association between smoking and SARS-CoV-2 infection risk. Smokers and non-smokers may differ in their health-seeking behaviour, including their likelihood of seeking medical care and testing for SARS-CoV-2. This could lead to a spurious negative association between smoking and the risk of infection. It is crucial to emphasise that the findings of this study should not be taken as an encouragement to start or continue smoking. Smoking is a well-established risk factor for numerous health issues, including lung cancer, heart disease and COPD. The low SARS-CoV-2 infection risk observed in this and other studies, even if causal, does not outweigh the negative health effects of smoking. Additional studies are needed to better understand the relationship between smoking and SARS-CoV-2 infection.

Our study has several strengths. First, the longitudinal study design allowed the study of the association of interest in a truly prospective fashion, avoiding the risk of reverse causality. Second, this study is population-based, hence minimising the risk of selection bias [34] present in hospital samples. Third, the rich database allowed a comprehensive adjustment for several potential confounders selected a priori according to causal pathways. Fourth, outcome assessment was done with a RT-PCR and registry-linked data, therefore, using information of high quality. Finally, the use of two data points to refine the exposure definition increased its sensitivity. Some limitations, however, should be noted. First, most information was based on self-reports. Second, the exposure assessment was conducted in 2014, long before the start of the COVID-19 pandemic. While this completely avoids reverse causation due to behavioural changes during the pandemic, it is likely that some individuals classified as current smokers in 2014 had subsequently quit smoking. In fact, the prevalence of daily smoking in the Swedish population has declined from 10% in 2014 to 7% in 2018–2020 [35]. However, if these misclassified former smokers had the same risk profile of the former smokers identified as such in this analysis, this would bias the negative association between current smoking and infection with SARS-CoV-2 rather towards the null.

Third, cohort participants, and particularly those retained between 2010 and 2014, may be a selected group with different behavioural and risk profile from those originally sampled. Caution should therefore be employed in extrapolating the results to the underlying source population. Also, we did not have information on occasional smoking; therefore occasional smokers are included in the ‘non-smoking’ group. Depending on assumptions on the risk profile of these smokers, the estimates of the relative risks of COVID-19 for former and current smokers may have been biased in either direction.

Former smokers in this study had a higher risk of COVID-19 infection and adverse disease outcomes. These findings indirectly support the public health efforts to curb smoking, given the effect of tobacco-related comorbidities. On methodologic grounds, separating non-, former, and current smokers when assessing the risk or the prognosis of diseases with an impact on the respiratory system emerges as an important suggestion.

## Supplemental Material

sj-docx-1-sjp-10.1177_14034948231174279 – Supplemental material for Does misclassification of former tobacco smokers explain the ‘smoker’s paradox’ in the risk of COVID-19? Insights from the Stockholm Public Health Cohort Click here for additional data file. Supplemental material, sj-docx-1-sjp-10.1177_14034948231174279 for Does misclassification of former tobacco smokers explain the ‘smoker’s paradox’ in the risk of COVID-19? Insights from the Stockholm Public Health Cohort by Ahmed N. Shaaban, Filip Andersson, Cecilia Magnusson, Nicola Orsini, Ida H. Caspersen, Sebastian Peña, Sakari Karvonen, Per Magnus and Maria R. Galanti in Scandinavian Journal of Public Health
