# Supplementary material for: Does misclassification of former tobacco smokers explain the ‘smoker’s paradox’ in the risk of COVID-19? Insights from the Stockholm Public Health Cohort
Source: Scand J Public Health. 2023 May 10;51(5):735–43. doi: 10.1177/14034948231174279 (PMC10183343; doi:10.1177/14034948231174279)

SUPPLEMENTARY MATERIAL

**Does misclassification of former tobacco smokers explain the “smoker's paradox” in the risk of COVID-19? Insights from the Stockholm Public Health Cohort**

# Supplementary Table S1. Crude Risk Ratios (RRs) and 95% Confidence Intervals (CI) of COVID-19 diagnosis and adverse outcomes for current smokers compared to non- or former smokers (non-current smokers).

|  |  | **Diagnoses of Covid-19** | **Hospital admission with COVID-19 diagnosis** | **Hospital admission with COVID-19 as the main diagnosis** | **Intensive care** | **Death due to COVID-19**  **as the main cause** | **Death within 30 days**  **with COVID-19 as the main or a contributory cause** |
| --- | --- | --- | --- | --- | --- | --- | --- |
|  |  | RR  (95% CI) | RR  (95% CI) | RR  (95% CI) | RR  (95% CI) | RR  (95% CI) | RR  (95% CI) |
| **Cigarette Smoking Status**  Non-smokers  Current smokers  Former smokers |  | (ref)  0.70 (0.61-0.80)  0.90 (0.85-0.96) | (ref)  1.15 (0.83-1.59)  1.52 (1.29-1.80) | (ref)  1.06 (0.74-1.53)  1.53 (1.27-1.83) | (ref)  0.94 (0.28-3.13)  1.57 (0.89-2.77) | (ref)   - 1. (0.52-1.96)   1.58 (1.15-2.18) | (ref)  1.16 (0.63-2.14)  1.63 (1.19-2.22) |

**Supplementary Figure S1. DAG of the hypothesized causal link between smoking and COVID-19.**

 
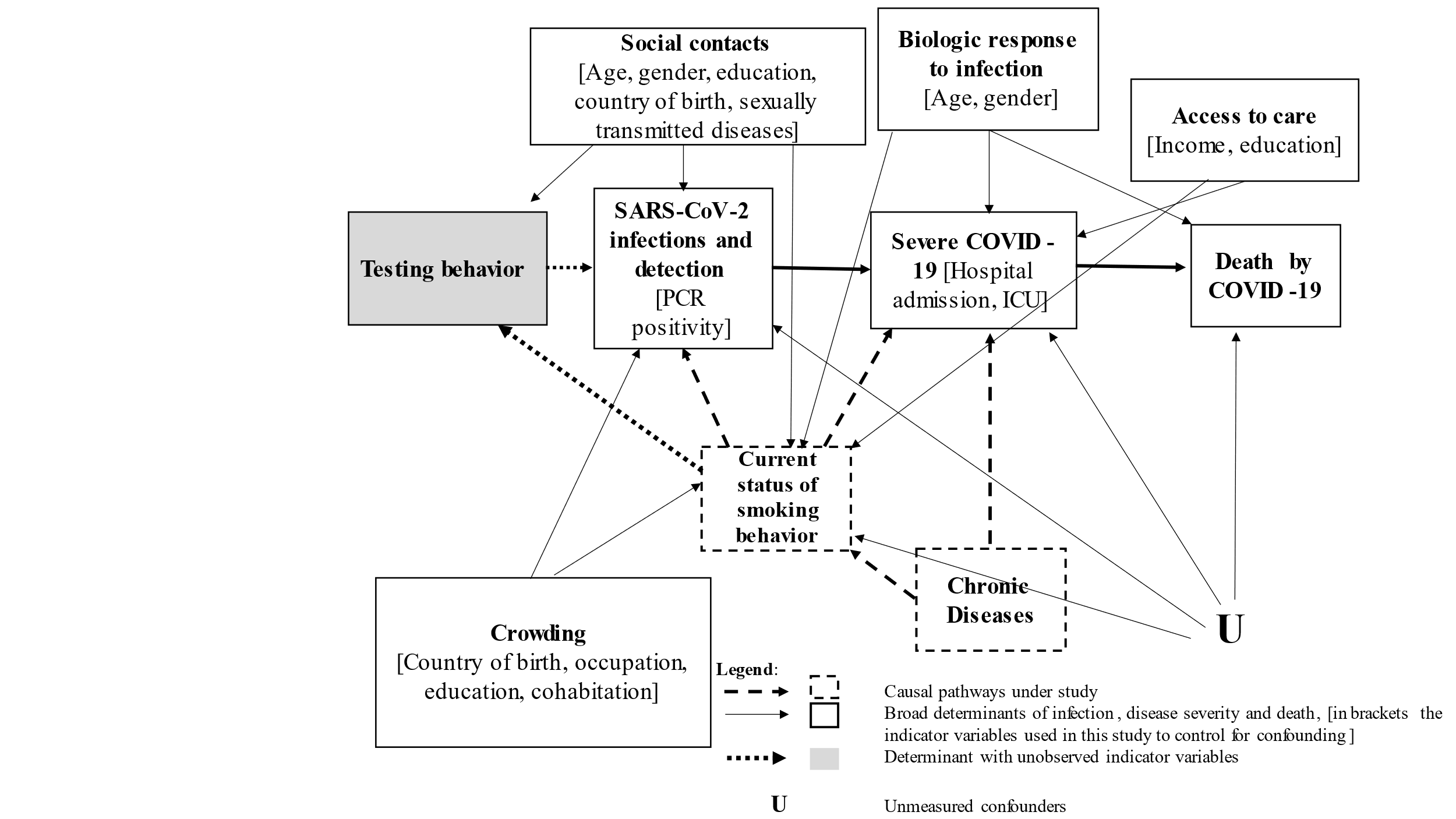

Supplement: sj-docx-1-sjp-10.1177_14034948231174279 – Supplemental material for Does misclassification of former tobacco smokers explain the ‘smoker’s paradox’ in the risk of COVID-19? Insights from the Stockholm Public Health Cohort [file sj-docx-1-sjp-10.1177_14034948231174279.docx]
